# Recognizing and understanding stress in adults during Covid-19: Data insights from the corona health app

**DOI:** 10.1016/j.dib.2025.111967

**Published:** 2025-08-12

**Authors:** Michael Winter, Thomas Probst, Dennis John, Rüdiger Pryss

**Affiliations:** a Institute of Medical Data Science, University Hospital Würzburg, Würzburg, Germany; b Institute of Clinical Epidemiology and Biometry, University of Würzburg, Würzburg, Germany; c Division of Psychotherapy, Department of Psychology, Paris Lodron University Salzburg, Salzburg, Austria; d Institute for Applied Research and Evaluation, Lutheran University of Applied Sciences, Nuremberg, Germany

**Keywords:** Covid-19, Ecological momentary assessment, Public health surveillance, Digital health monitoring, Perceived stress scale

## Abstract

The dataset presented in this work is derived from the Stress Recognition Study in the Corona Health app, a digital health platform designed with the German Robert Koch Institute (RKI) to monitor stress levels and associated factors in adults during and after the COVID-19 pandemic. Data were collected using a mobile-based survey completed by 627 adults (18 years and older) at baseline, with 385 of these participants also contributing 4,331 follow-up assessments over time. The study utilized baseline and follow-up questionnaires to capture changes in participants' stress levels throughout the pandemic period and beyond (December 2020 to May 2025). The questionnaires cover key stress indicators such as perceived stress levels, demographic factors, and smartphone sensor data. By capturing real-time, longitudinal stress data from adults during a public health crisis, this dataset enables researchers to examine how stress levels fluctuated in response to pandemic restrictions and recovery phases. The integration of ecological momentary assessments with mobile sensing data (e.g., app usage statistics, coarse-grained location information) provides opportunities to analyze adult stress trajectories, identify stress resilience factors, and evaluate the effectiveness of mobile health approaches for stress monitoring during crisis situations. The data, including questionnaire responses and mobile sensing data, are publicly available under a Creative Commons license at https://zenodo.org/records/15780255.

Specifications Table

**Table:** 

Subject	Health Sciences, Medical Sciences & Pharmacology
Specific subject area	Smartphone-based stress assessments of adults during COVID-19
Type of data	Table, Figure, Raw
Data collection	The Corona Health platform facilitated data collection through self-reported surveys, incorporating Ecological Momentary Assessments (EMA) and Patient-Reported Outcome Measures (PROMs). It captured structured questionnaire responses and passive sensor data, such as app usage patterns. The questionnaires, adapted from validated instruments, were translated into eight languages.
Data source location	Most of the data originated in Germany (98.09 %). The data source is the Institute of Clinical Epidemiology and Biometry at the University of Würzburg, located in Würzburg, Germany (49.7882° N, 9.9353° E).
Data accessibility	Repository name: Zenodo DOI: 10.5281/zenodo.15780254 Direct URL to data: https://zenodo.org/records/15780255
Related research article	none

## 1 Value of the Data

• The data collected in the Corona Health Stress Recognition Study provide valuable insights into adult stress levels and related mechanisms, particularly in the context of the COVID-19 pandemic and its aftermath from December 2020 until May 2025 [1]. Utilizing Ecological Momentary Assessment (EMA) and mobile sensing technologies, this dataset captures real-time self-reports on perceived stress using the validated Perceived Stress Scale (PSS-10) [2]. By integrating baseline demographic data and weekly follow-up assessments, the data enables the study of stress trajectories and associated factors (e.g., age, marital status, COVID-19 exposure) affecting adults over an extended pandemic period [14].
• This dataset allows for an in-depth analysis of adult stress perception during a global health crisis. It includes a broad spectrum of variables, such as demographic characteristics, COVID-19-related experiences (e.g., infection status, affected relatives, losses), smartphone usage patterns, and location data, offering various perspectives on stress determinants. The study's smartphone-based methodology ensures high ecological validity, reducing recall bias, and increasing accessibility for participants who might be less likely to engage in traditional stress assessment methods.
• While the dataset is based on a convenience sample, its comprehensive scope and methodological rigor provide valuable opportunities for exploring patterns of adult stress responses and resilience factors during pandemic conditions. This was ensured by relying on the validated PSS-10 instrument, multilingual availability, notifications, and standardized digital protocols. The structured weekly data collection process and ethical compliance enhance its applicability for stress research, intervention development, and public health initiatives.
• This dataset offers opportunities for longitudinal analyses of adult stress trajectories during and after pandemic restrictions, spanning from December 2020 to May 2025. Researchers can examine how perceived stress levels fluctuated in response to changing pandemic policies, lockdown measures, economic uncertainties, and social restrictions. The inclusion of follow-up data from 385 participants contributing 4,331 assessments allows for the investigation of stress patterns over time, providing insights into which adults maintained low stress levels versus those who experienced prolonged stress effects throughout different pandemic phases.
• The dataset's documentation and structured format enable diverse methodological approaches, supporting methods ranging from time-series analysis to machine learning approaches for stress prediction models. Researchers can utilize the standardized PSS-10 assessment tool to conduct comparative analyses with other adult stress datasets or population studies. Additionally, the combination of self-reported stress questionnaires and mobile sensing data offers unique possibilities for examining relationships between subjective stress experiences and objective behavioral indicators, potentially informing the development of more responsive digital stress monitoring and intervention systems for adults in crisis situations.

## 2 Background

The COVID-19 pandemic presented challenges to global mental health, particularly affecting stress levels across diverse populations [3]. Recognizing the need for real-time monitoring of psychological well-being during this crisis, we developed Corona Health, a comprehensive digital health surveillance platform in collaboration with the German Robert Koch Institute [1]. An objective of this platform aimed to capture stress experiences among adults as they were confronted with the prolonged challenges of the pandemic.

Traditional stress assessment methods, typically relying on retrospective self-reports collected at discrete time points, proved inadequate for understanding the dynamic nature of stress responses during rapidly changing circumstances [4]. To address these limitations, we implemented a mobile-based approach that integrated multiple data collection methodologies to assess daily fluctuations of stress levels [14]. In more detail, Corona Health incorporated Ecological Momentary Assessment (EMA) techniques [5], which capture real-time experiences in participants' natural environments, alongside Patient-Reported Outcome Measures (PROMs) [6] for standardized assessment protocols.

The integration of Mobile Crowdsensing [7] and Digital Phenotyping [8] technologies enabled the collection of objective behavioral indicators that complement subjective stress reports. This chosen approach provides insights into the relationship between self-reported stress experiences and observable behavioral patterns, such as mobility changes and smartphone usage variations [15].

Our longitudinal study design spans from December 2020 through May 2025. The study recruited 627 adults who completed comprehensive baseline assessments, with 385 participants providing sustained engagement through 4,331 follow-up evaluations. This extensive longitudinal dataset enables examination of stress adaptation patterns, identification of resilience factors, and analysis of how different demographic groups responded to evolving pandemic conditions.

Central to our methodology is the implementation of the Perceived Stress Scale (PSS-10) [2], a validated instrument with sound psychometric properties that ensures scientific consistency. PSS-10 enables comparative analysis with existing stress research literature. The weekly assessment protocol, combined with passive data collection through mobile sensors, creates a rich dataset for understanding immediate stress responses and longer-term adaptation processes.

This research addresses existing gaps in understanding adult stress dynamics during global health emergencies, providing evidence for developing more responsive mental health interventions and informing public health policy decisions for future crisis preparedness.

## 3 Data Description

The complete dataset and documentation are made publicly available under a Creative Commons license at https://zenodo.org/records/15780255 [16]. Detailed specifications for all survey questions and response formats are presented in Table 2, while comprehensive variable definitions and coding schemes are provided in the accompanying documentation available through the data repository. The repository contains unprocessed raw data files and analysis-ready processed versions, including baseline stress assessments (answersheets_stress_baseline.csv) and follow-up evaluations (answersheets_stress_followUp.csv). The contextual sensing data encompasses technical specifications outlined in Table 3, including hardware information, geographic coordinates, and sensor metadata captured during assessment periods. Standardized data structures and uniform variable nomenclature across all files enable seamless integration for comprehensive longitudinal stress analysis spanning the entire study duration.

### 3.1 Survey instruments and content

The Stress Recognition Study implemented through the Corona Health platform encompasses two primary assessment instruments: an initial comprehensive questionnaire and recurring evaluation surveys. Furthermore, upon obtaining participant authorization, passive mobile data collection was integrated to enhance the self-documented information provided by users.

### 3.2 Baseline questionnaire

The baseline questionnai re starts with a section collecting essential demographic information from participants, including age, gender, country of residence, marital status, and educational background. The complete survey encompasses 19 questions organized into the following thematic areas:

• Demographic and Personal Information: These questions gather fundamental participant characteristics such as age, gender, marital status, educational attainment, and country of residence, establishing the foundational profile for each respondent.
• COVID-19 Experience and Impact: Questions examining participants' direct and indirect experiences with COVID-19, including personal infection status, family member illness, and potential losses due to the pandemic, providing context for stress experiences.
• Perceived Stress Assessment: A comprehensive 10-item evaluation utilizing the validated Perceived Stress Scale (PSS-10), measuring participants' subjective stress experiences. To better capture the dynamics of perceived stress, we changed the instruction to evaluate the stress-level with regard to the timeframe of the current week instead of the past month. These questions assess feelings of being overwhelmed, confidence in handling problems, ability to control life situations, and perceptions of difficulties accumulating beyond one's capacity to manage them.

### 3.3 Follow-up questionnaire

The follow-up questionnaire reassesses solely the Perceived Stress Scale. It specifically focuses on capturing temporal variations in stress patterns, facilitating comprehensive analysis of stress level trajectories throughout the extended study duration (see Table 2, Column Follow-Up?).

### 3.4 Mobile sensing data

The Corona Health platform gathered passive mobile data, when consent from participants was given. This information includes technical device specifications, coarse-grained geographic coordinates captured during survey completion sessions, and summarized smartphone application usage metrics (restricted to Android operating systems).

### 3.5 Dataset overview

**Fig. 1:**
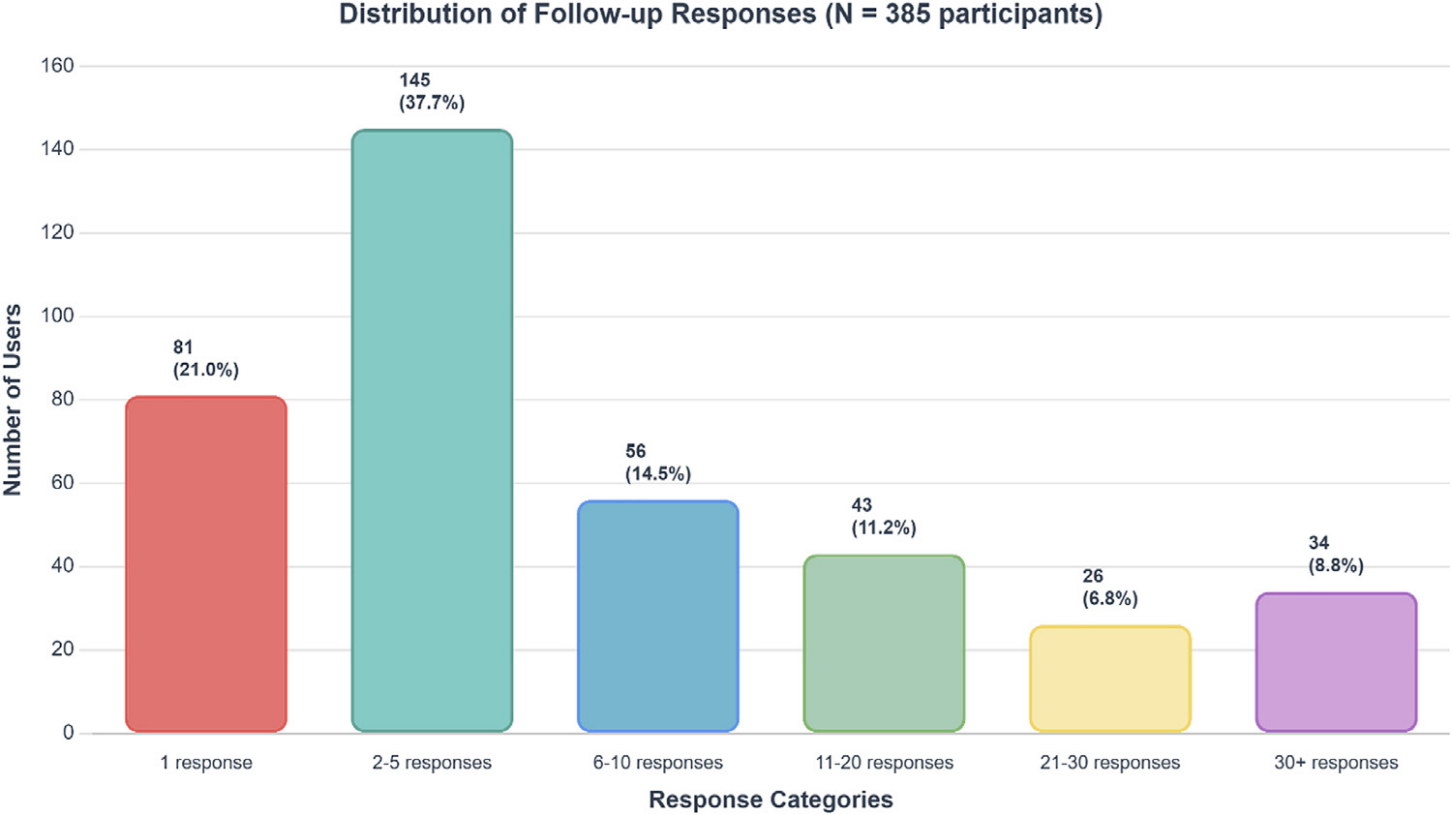
Number of follow-up responses by the participants.

The dataset encompasses responses from initial assessments and subsequent longitudinal evaluations gathered between December 2020 and May 2025. Additionally, the dataset incorporates geographic positioning information and digital behavioral metrics. The collection of digital phenotyping data was exclusively available for Android device users. Specifically, the repository stores mobile operating system details, Corona Health application versions, device specifications, smartphone usage patterns, and geographic coordinate information (see Table 2, Table 3). Table 1 summarizes the baseline demographics of the 627 study participants (statistics shown in the following were determined in Microsoft Excel). The gender composition demonstrates a female predominance (64.43% vs. 34.29% male vs. 1.28 % transgender), with a mean participant age of 40.72 years (SD = 13.97). The majority utilized Android devices (84.21%) compared to iOS users (15.79%). Regarding COVID-19 exposure, 88.52% of participants reported no positive test results, while 11.48% confirmed previous infection. Most of the participants (98.09 %) were located in Germany during their initial questionnaire completion Fig. 1.

**Table 1:** Demographics and key numbers about the baseline questionnaire.

No. Questionnaires	627 (users) – Baseline 4331 – Follow-up - 385 users
No. Tracking GPS	440 (70.17 %)
No. Tracking App Usage	58 (9.25 %)
mobile OS	528 (84.21 %) – Android 99 (15.79 %) - iOS
Age (mean (SD), in years)	40.88 (14.30)
Gender	404 (64.43 %) – Female 215 (34.29 %) – Male 8 (1.28 %) – Transgender
Marital Status	351 (55.98 %) – Married or solid partnership 12 (1.91 %) – Married, living apart 43 (6.86 %) – Divorced 1 (0.16 %) – Registered civil partnership (same-sex) 0 (0.0 %) – Registered civil partnership (same-sex), living apart 9 (1.44 %) – Widowed 211 (33.65 %) – Single
COVID-19 positive?	555 (88.52 %) – No 72 (11.48 %) - Yes
PSS item 3: felt nervous and ``stressed''	33 (5.26 %) – Never 76 (12.12 %) – Almost never 216 (34.45 %) – Sometimes 205 (32.70 %) – Fairly often 97 (15.47 %) – Very often

**Table 2:** Detailed description of the baseline and follow-up questionnaire. Because these questionnaires share a major part of their questions, we provide these here within one table.

Question Type	Label	Follow-Up?	Question	Encoding and Answer Options
SingleChoice	pers	No	Do you fill out the questionnaire for yourself or another person?	1 = For myself, 2 = For another person
Knob	age	No	How old are you (in years)?	Min = 18, Max = 120, Step = 1
SingleChoice	gender	No	Which gender are you?	0 = Female, 1 = male, 2 = Transgender
SingleChoiceKnob	country	No	In which country do you currently live?	
SingleChoice	family	No	What is your marital status?	1 = Married or solid partnership, 2 = Married, living apart, 3 = Divorced, 4 = Registered civil partnership, 5 = Registered civil partnership, living apart, 6 = Widowed, 7 = Single
SingleChoice	education	No	How many years have you been in school in total?	1 = 7 or less, 2 = 8 to 9, 3 = 10, 4 = 11 to 12, 5 = 13 or more, 6 = I am still at school
SingleChoice	covid1	No	Have you been tested positive for COVID-19?	0 = No, 1 = yes, currently ill, 2 = Yes, already recovered
SingleChoice	covid2	No	Do you have any relatives infected with COVID-19?	0 = No, 1 = yes, currently ill, 2 = Yes, already recovered
YesNoSwitch	covid3	No	Have you lost relatives or friends due to COVID-19?	0 = No, 1 = Yes
SingleChoice	pss1	Yes	In the last week, how often have you been upset because of something that happened unexpectedly?	0 = Never, 1 = Almost never, 2 = Sometimes, 3 = Fairly often, 4 = Very often
SingleChoice	pss2	Yes	In the last week, how often have you felt that you were unable to control the important things in your life?	0 = Never, 1 = Almost never, 2 = Sometimes, 3 = Fairly often, 4 = Very often
SingleChoice	pss3	Yes	In the last week, how often have you felt nervous and “stressed”?	0 = Never, 1 = Almost never, 2 = Sometimes, 3 = Fairly often, 4 = Very often
SingleChoice	pss4	Yes	In the last week, how often have you felt confident about your ability to handle your personal problems?	0 = Never, 1 = Almost never, 2 = Sometimes, 3 = Fairly often, 4 = Very often
SingleChoice	pss5	Yes	In the last week, how often have you felt that things were going your way?	0 = Never, 1 = Almost never, 2 = Sometimes, 3 = Fairly often, 4 = Very often
SingleChoice	pss6	Yes	In the last week, how often have you found that you could not cope with all the things that you had to do?	0 = Never, 1 = Almost never, 2 = Sometimes, 3 = Fairly often, 4 = Very often
SingleChoice	pss7	Yes	In the last week, how often have you been able to control irritations in your life?	0 = Never, 1 = Almost never, 2 = Sometimes, 3 = Fairly often, 4 = Very often
SingleChoice	pss8	Yes	In the last week, how often have you felt that you were on top of things?	0 = Never, 1 = Almost never, 2 = Sometimes, 3 = Fairly often, 4 = Very often
SingleChoice	pss9	Yes	In the last week, how often have you been angered because of things that were outside of your control?	0 = Never, 1 = Almost never, 2 = Sometimes, 3 = Fairly often, 4 = Very often
SingleChoice	pss10	Yes	In the last week, how often have you felt difficulties were piling up so high that you could not overcome them?	0 = Never, 1 = Almost never, 2 = Sometimes, 3 = Fairly often, 4 = Very often

**Table 3:** Detailed description of additional collected contextual data.

Column Name	Description
id	Unique identifier for each record.
user_id	Identifier for the user who generated the data.
collected_at	Timestamp indicating when the data was collected.
client_os	Operating system of the client device that collected the data.
client_device	Specific device name or type of the client device.
sensordata_altitude	Altitude data from the sensor at the time of collection (e.g., elevation above sea level).
sensordata_name	Name or type of the sensor that collected the data.
sensordata_collected_at	Timestamp when the sensor data was collected.
sensordata_longitude	Longitude coordinate of the sensor at the time of data collection.
sensordata_latitude	Latitude coordinate of the sensor at the time of data collection.

**Fig. 2:**
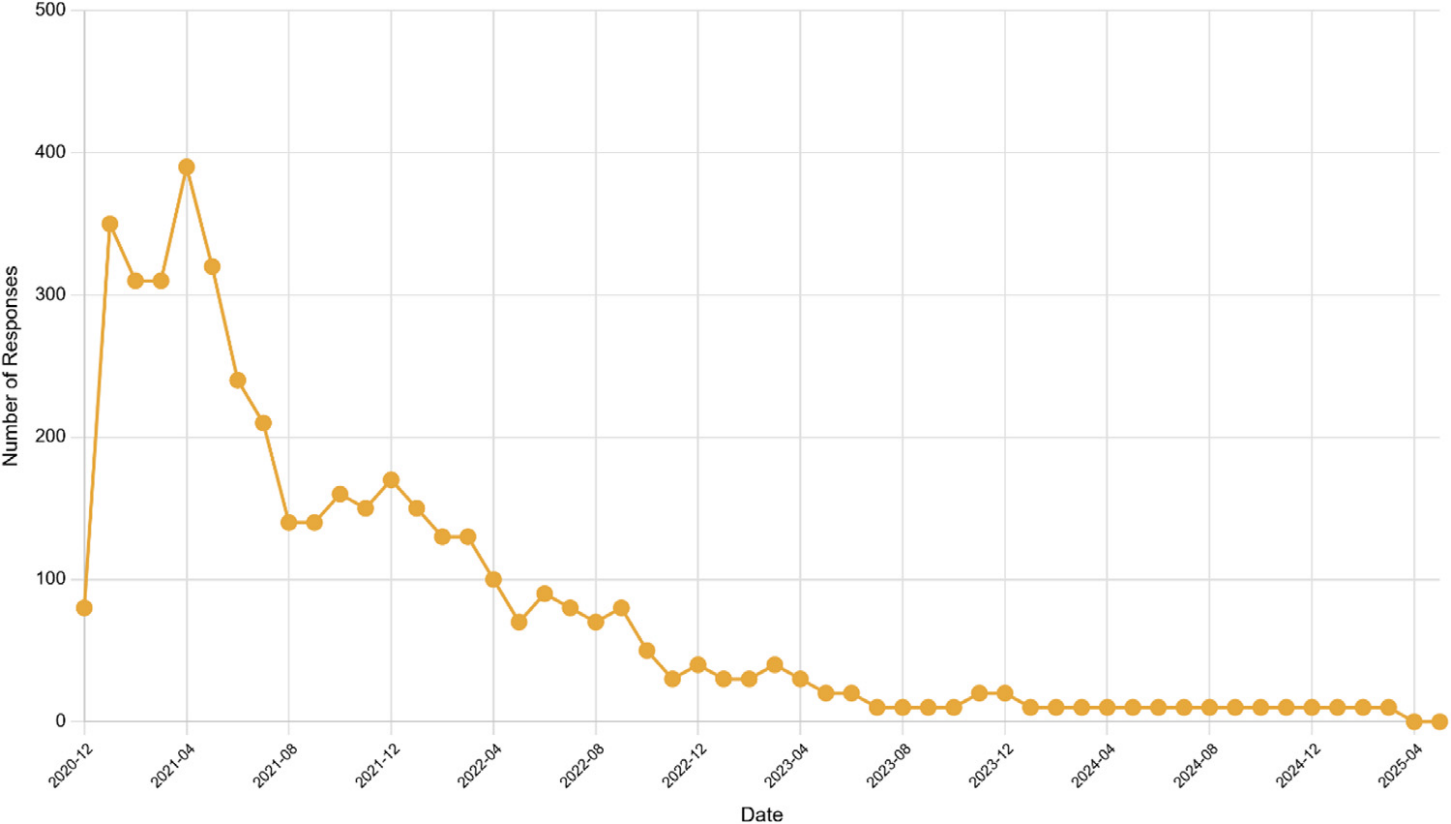
Number of follow-up responses over time.

A total of 385 participants provided both initial assessments and subsequent follow-up evaluations. Response frequency per individual demonstrated considerable variability, ranging from single contributions to a maximum of 216 responses from one participant. The participation distribution (see Fig. 2) reveals differences in user engagement levels, reflecting typical challenges encountered in extended digital health monitoring studies [13]. Such engagement patterns are characteristic of longitudinal research designs, where a dedicated minority of participants generates a disproportionate share of the overall response data.

**Fig. 3:**
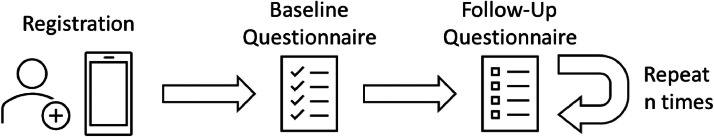
Study workflow in Corona health

While the intended interval between follow-up responses was approximately one week, the actual observed average interval was 11.20 days. Analysis of 4,331 response intervals revealed that 60.1 % of responses fell within the target 4 - 7 days range, while 14.4 % occurred within 8-14 days, indicating that 74.5 % of all follow-up assessments maintained reasonable adherence to the weekly protocol. Such variability in response timing must be carefully considered in subsequent analyses to ensure the robustness of findings. Additionally, participant engagement followed a declining pattern over the 54-month study period, with peak activity occurring in early 2021 (i.e., 386 responses in April) before gradually decreasing to low-level engagement by 2023-2025, where a dedicated minority of individuals contributed to a disproportionately high number of responses (see Fig. 2, Fig. 3). Comparable trends in participant engagement have been observed in previous studies conducted as part of the Corona Health project [9].

Regarding the geospatial distribution of participants within Germany, analysis of the 440 participants who provided GPS tracking data (70.2 % of all participants) revealed that engagement was highest in Baden-Württemberg (105 participants, 24.5 %), followed by Bayern (88 participants, 20.6 %) and Niedersachsen (68 participants, 15.9 %). The study achieved geographic coverage across 15 of Germany's 16 federal states, with only Saarland having no participants in the GPS-enabled dataset. Southern German states demonstrated particularly strong participation, with Baden-Württemberg and Bayern together accounting for 45.1 % of all GPS-tracked participants, while eastern states showed lower but meaningful representation, ranging from 2 participants in Sachsen-Anhalt (0.5 %) to 18 participants in Brandenburg (4.2 %).

The geographic disparities in participation rates reflect patterns commonly encountered in digital health research, where states with larger populations typically demonstrate greater user engagement. Concurrently, the concentration phenomenon observed, wherein a dedicated minority of users generated substantially more data than the majority, mirrors results documented in comparable digital health investigations [10]. These engagement characteristics highlight the critical need to account for territorial participation imbalances and varying levels of individual commitment when analyzing findings, as such factors can impact the generalizability and methodological integrity of longitudinal research outcomes.

It is essential to outline the temporal framework of our data acquisition methodology. Although the Corona Health platform employed EMA techniques, our approach incorporated reduced sampling intervals compared to conventional high-frequency EMA frameworks. The research protocol aimed for weekly assessment cycles to optimize the balance between data comprehensiveness and sustained user participation throughout the prolonged investigation period (December 2020 to May 2025).

The variance from the planned weekly assessment rhythm resulted from multiple contributing elements. Initially, the participation model ensured that compliance remained user-controlled rather than mandated through rigid notification protocols. Furthermore, the protracted pandemic timeline and corresponding research duration presumably fostered engagement decline, a frequently encountered obstacle in extended digital health investigations. Our team intentionally avoided implementing excessive alert mechanisms that could have enhanced compliance but risked elevating discontinuation rates among participants. Although this data collection strategy yielded reduced temporal precision relative to intensive EMA methodologies, it effectively documented significant longitudinal patterns in adult stress experiences while preserving adequate participant involvement across the multi-year research timeline.

## 4 Experimental Design, Materials and Methods

This section presents a brief outline of the application's creation, study methodology, information gathering procedures, ethical protocols, and technological infrastructure.

### 4.1 Application design and implementation

The Corona Health platform, constructed utilizing the TrackYourHealth platform [11], provided multilingual accessibility in eight languages. The creation process encompassed cross-disciplinary partnerships, conformity with regulatory mandates, and incorporation of validated approaches to guarantee data integrity and adherence to ethical protocols. The platform deployed five studies related to physical and mental well-being (e.g., mental health in adolescents [12]).

The platform was engineered according to EU Medical Device Regulation specifications, securing robust privacy safeguards and participant protection. To strengthen data precision, Ecological Momentary Assessment (EMA) methodologies were deployed, reducing retrospective reporting errors. Furthermore, sensor-driven and digital behavioral tracking methods enabled the acquisition of objective measurements, including geographic coordinates, to complement participant-reported information.

### 4.2 Data acquisition and collection process

The Corona Health application functioned as a digital interface for gathering ecological momentary evaluations (EMA) and sensor information concerning physical health and psychological well-being throughout the COVID-19 crisis. The user experience operated as follows:

Following application download and installation, users initially encountered a consent disclaimer that required approval before proceeding. After providing consent, users could explore and enroll in any of five research investigations (adolescent mental health, adult mental health, adult physical health, stress assessment, and COMPASS initiative [1]), with the Stress Recognition Study constituting the focus of this manuscript.

In the Stress Recognition Study, users completed an initial comprehensive assessment (10-15 minutes duration) that documented their baseline stress level profile. During this first evaluation, users received requests for authorization to gather positional information (across both Android and iOS platforms) and application usage metrics (Android devices exclusively). Geographic data was intentionally configured with reduced precision (11.1 km resolution) to ensure participant anonymity.

Subsequent to the initial evaluation, the platform generated scheduled recurring assessments every week (approximately 5 minutes) according to the specific study protocol (see Figure 4). Users received automated prompts for these evaluations while retaining the option to complete them independently at their convenience. Response data was cached locally during offline periods and transmitted to the server upon connectivity restoration.

The collected sensor metrics encompassed:

• Hardware specifications and operating system details
• Approximate geographic position during assessment completion (when authorized)
• For Android users with granted permissions: compiled application usage analytics including daily device engagement, active/inactive intervals, and social media platform statistics

All gathered information underwent anonymization and was archived in a systematized database for research applications, with personalized feedback delivered to users through the application's information interface based on their submission patterns.

### 4.3 Survey workflow and data processing

The survey instrument creation workflow commenced with question design in Microsoft Excel, establishing metadata specifications, response formats, and multilingual configurations. These documents were then transformed into JSON structure through Python scripting and incorporated into the server infrastructure, providing access via a RESTful API framework. This methodology enabled flexible implementation, supporting immediate modifications throughout the application environment. Information transfer utilized JSON protocols, guaranteeing systematic and streamlined procedures for both survey deployment and user response processing.

The platform became available in eight languages (i.e., German, English, Spanish, French, Hungarian, Italian, Russian, Slovenian through Google Play and Apple App Store distributions. During initial application launch, users encountered mandatory consent documentation followed by configuration guidance covering notification settings and privacy authorizations. The Corona Health architecture was constructed to accommodate both connected and disconnected operations, maintaining uninterrupted data acquisition capabilities. Participant information was archived and managed through a relational SQL infrastructure, preserving data consistency and organized administration.

## Limitations

Participant discontinuation substantially impacts longitudinal evaluations, with 385 (61.4 %) of original users supplying follow-up information. The markedly diverse participation behaviors, extending from individual submissions to exceeding 200 follow-ups from particular users, produces methodological complications for investigating longitudinal developments of stress levels.

The spatial and chronological irregularities in data gathering introduce further analytical difficulties. In more detail, the restricted GPS authorization rate, with 440 participants (70.2 %) supplying positional information, constrains the geographical validity of spatial investigations and potentially introduces selection bias in territorial comparisons. These imbalances in engagement across German regions and the divergence from planned weekly evaluation cycles (documented mean: 10.41 days) demand statistical attention when investigating geographic distributions or chronological patterns in adult stress measurements.

The study sample shows a significant imbalance in mobile operating systems, with 528 (84.21 %) of participants using Android devices compared to only 99 (15.79 %) using iOS devices. This disproportionate representation may limit the generalizability of findings across different smartphone user populations. Further, mobile sensing data collection, particularly smartphone application usage metrics, was restricted exclusively to Android devices due to platform limitations, potentially limiting comprehensive digital phenotyping analyses and reducing the dataset's utility for examining relationships between smartphone behavior and stress levels.

Additionally, the non-probabilistic recruitment strategy and elective participation framework may restrict the transferability of results to wider adult demographics, as users presumably represent individuals with elevated technological proficiency and heightened interest in stress surveillance throughout the pandemic period.

## Ethics Statement

This study adhered to the German Medical Device Regulations (MDR), the General Data Protection Regulation (GDPR), and the principles of the Declaration of Helsinki. Data collection was performed via the Corona Health app. Approval for this study was granted by the Ethics Committee and the data protection officer of the University of Würzburg, Germany (approval no. 130/20-me). Participants provided general consent for app use and specific consent for GPS-based location tracking and digital phenotyping data. All data were anonymized, and rigorous data protection measures were employed to ensure participant privacy throughout the study.

## CRediT authorship contribution statement

**Michael Winter:** Conceptualization, Methodology, Software, Data curation, Formal analysis, Visualization, Writing – original draft, Writing – review & editing. **Thomas Probst:** Writing – review & editing, Validation. **Dennis John:** Writing – review & editing, Supervision. **Rüdiger Pryss:** Supervision, Project administration, Conceptualization, Writing – review & editing, Funding acquisition.

## Data Availability

ZenodoUnderstanding Stress in Adults During COVID-19 (Original data)
